# BAMBI Promotes C2C12 Myogenic Differentiation by Enhancing Wnt/β-Catenin Signaling

**DOI:** 10.3390/ijms160817734

**Published:** 2015-08-03

**Authors:** Qiangling Zhang, Xin-E Shi, Chengchuang Song, Shiduo Sun, Gongshe Yang, Xiao Li

**Affiliations:** Laboratory of Animal Fat Deposition and Muscle Development, College of Animal Science and Technology, Northwest A&F University, Yangling 712100, China; E-Mails: langyan.95@163.com (Q.Z.); xineshi@nwsuaf.edu.cn (X.-E.S.); chengchuangsong@163.com (C.S.); ssdsm@tom.com (S.S.); gsyang999@hotmail.com (G.Y.)

**Keywords:** BAMBI, Wnt/β-catenin, myogenic differentiation, LiCl, C2C12

## Abstract

Bone morphogenic protein and activin membrane-bound inhibitor (*BAMBI*) is regarded as an essential regulator of cell proliferation and differentiation that represses transforming growth factor-β and enhances Wnt/β-catenin signaling in various cell types. However, its role in skeletal muscle remains largely unknown. In the current study, we found that the expression level of *BAMBI* peaked in the early differentiation phase of the C2C12 rodent myoblast cell line. Knockdown of *BAMBI* via siRNA inhibited C2C12 differentiation, indicated by repressed *MyoD*, *MyoG*, and *MyHC* expression as well as reductions in the differentiation and fusion indices. BAMBI knockdown reduced the activity of Wnt/β-catenin signaling, as characterized by the decreased nuclear translocation of β-catenin and the lowered transcription of *Axin2*, which is a well-documented target gene of the Wnt/β-catenin signaling pathway. Furthermore, treatment with LiCl, an activator of Wnt/β-catenin signaling, rescued the reduction in C2C12 differentiation caused by *BAMBI* siRNA. Taken together, our data suggest that *BAMBI* is required for normal C2C12 differentiation, and that its role in myogenesis is mediated by the Wnt/β-catenin pathway.

## 1. Introduction

Bone morphogenic protein and activin membrane-bound inhibitor (*BAMBI*) is a transmembrane protein composed of 261 amino acids in mice, which shares high similarity with the transforming growth factor-β (TGF-β) family type I receptor in their extracellular domains, while lacking an intracellular kinase domain [1,2]. *BAMBI* has been reported to promote the invasion, migration, and proliferation of cancer cells [3,4], and to suppress adipogenesis [5,6]. Previous studies have shown that *BAMBI* significantly inhibits the expression of carcinoma-associated fibroblast markers in human bone marrow mesenchymal stem cells without affecting their stem cell and tumor-tropic properties [7].

*BAMBI* is involved in several signaling pathways. *BAMBI* can interfere with the association between the TGF-β type I and type II receptors [1] and inhibit the interaction between TGF-β receptor I and *Smad3* as a decoy receptor in mouse embryonic carcinoma P19 cells and human embryonic kidney HEK293T cells [2], respectively. *BAMBI* also promotes the activity of Wnt/β-catenin signaling in porcine preadipocytes [5], HEK293T cells [8], and Simpson–Golabi–Behmel syndrome preadipocytes [6]. Moreover, *BAMBI* has been proposed to mediate the inductive effect of fibroblast growth factor on the expression of the sonic hedgehog gene during limb regeneration [9].

Wnt/β-catenin signaling plays an essential role during embryonic muscle development and adult skeletal muscle homeostasis. Wnt signaling activity promotes the expansion and differentiation of myogenic progenitors during lineage specification and muscle regeneration [10]. *R-spondins*, which have been characterized as Wnt/β-catenin signaling activators, promote myogenesis and induce hypertrophic myotube formation in C2C12 cells [11,12]. Glycogen synthase kinase-3β is a negative regulator of Wnt/β-catenin signaling, and inhibiting this kinase enhances the myogenic differentiation of C2C12 cells [13,14].

Given that *BAMBI* is involved in regulating the proliferation and differentiation of multiple cell types, and that Wnt/β-catenin signaling is essential for myogenic differentiation, we proposed that *BAMBI* might influence C2C12 differentiation, and that Wnt/β-catenin signaling might also be involved. In our studies, we blocked *BAMBI* expression using siRNA and found that *BAMBI* plays essential roles in C2C12 myogenic differentiation and Wnt/β-catenin signaling transduction.

## 2. Results

### 2.1. Expression Patterns of BAMBI

**Figure 1:**
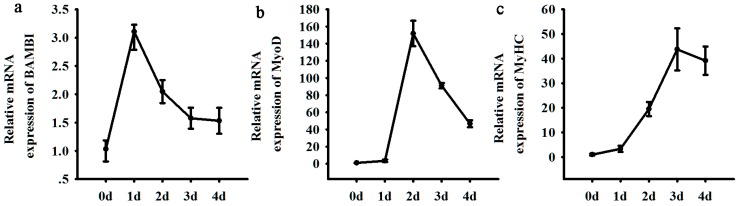
The temporal mRNA expression profiles of *BAMBI* (**a**), *MyoD* (**b**) and *MyHC* (**c**) during C2C12 myogenic differentiation. The results were represented as mean ± SD.

Our data showed that *BAMBI* mRNA expression increased three-fold on day 1 of myogenesis compared with its expression in undifferentiated cells on day 0, and then gradually declined in the following days (Figure 1a). The expression of MyoD, a well-known myogenic marker, peaked on day 2 post differentiation (Figure 1b). The peak of *BAMBI* expression was observed prior to that of *MyoD* expression, indicating that BAMBI might play a role during the early phase of C2C12 differentiation. The differentiation status of C2C12 cells was monitored by measuring the expression levels of *MyoD* and *MyHC*, which both consistently increased over time (Figure 1b,c).

### 2.2. Knockdown of BAMBI Inhibits C2C12 Myogenic Differentiation

**Figure 2:**
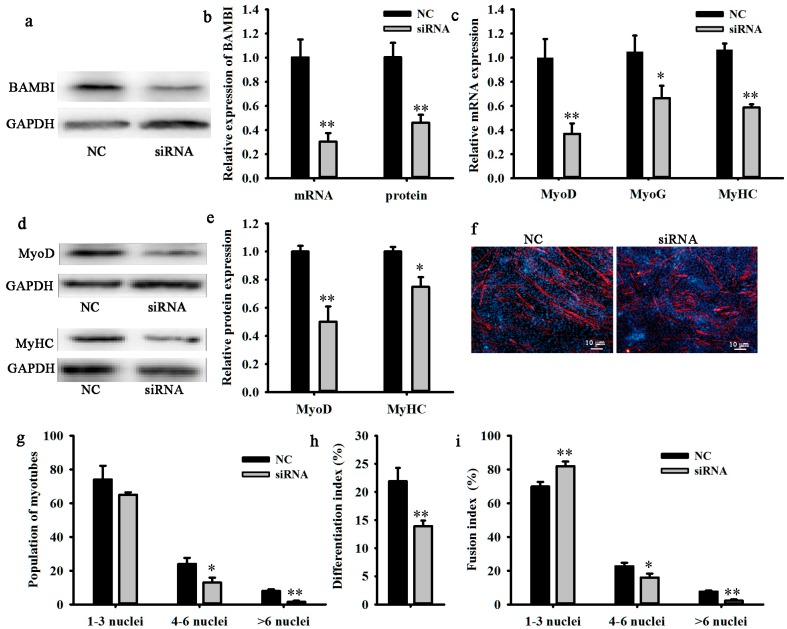
Knockdown of BAMBI inhibited myogenic differentiation. All the cell samples were harvested after transfection and myogenic induction for 48 and 96 h. (**a**) The western blot images of BAMBI and GAPDH; (**b**) the efficiency of siRNA interference on the mRNA and protein expression of *BAMBI*; (**c**) the mRNA expression of *MyoD* at 48 h and that of *MyoG* and *MyHC* at 96 h; (**d**) the western blot images of MyoD at 48 h, MyHC at 96 h, and their corresponding GAPDH; (**e**) the protein expression of MyoD at 48 h and MyHC at 96 h; (**f**) immunofluorescence of MyHC in C2C12 myotubes at 96 h post differentiation, images captured at 100× magnification; (**g**) the populations of myotubes; (**h**) the differentiation index; and (**i**) the myotube fusion index. The results were represented as mean ± SD; *n* = 3; * *p* < 0.05; ** *p* < 0.01.

To explore the role of BAMBI during C2C12 myogenic differentiation, siRNA was used to interfere with the expression of *BAMBI*. Results demonstrated that *BAMBI* siRNA significantly inhibited both the mRNA and protein expression of *BAMBI* at 48 h post transfection and induction (Figure 2a,b). Meanwhile, the expression levels of *MyoD* at 48 h as well as those of *MyoG* and *MyHC* at 96 h were significantly suppressed (Figure 2c–e). The population of myotubes was obviously reduced, especially in the myotubes with 4–6 nuclei and >6 nuclei (Figure 2f,g). The differentiation and fusion indices were also decreased (Figure 2h,i). These results suggest that the interference of *BAMBI* expression inhibits the differentiation of C2C12 cells.

### 2.3. Knockdown of BAMBI Inhibits Wnt/β-Catenin Signaling during C2C12 Differentiation

**Figure 3:**
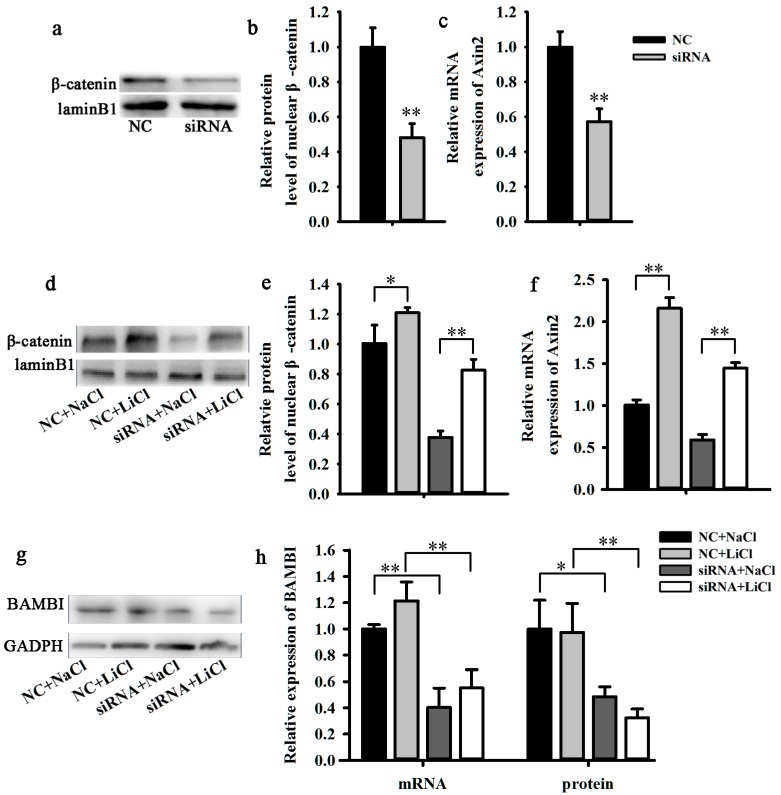
Knockdown of *BAMBI* suppressed Wnt/β-catenin signaling. All the cell samples were harvested after transfection and myogenic induction for 48 h. (**a**) The western blot images of nuclear β-catenin and laminB1; (**b**) the nuclear β-catenin protein levels; (**c**) the mRNA expression of Axin2; (**d**) the western blot images of nuclear β-catenin and laminB1; (**e**) the nuclear β-catenin protein levels; (**f**) the mRNA expression of Axin2; (**g**) the western blot images of BAMBI and GAPDH; and (**h**) the mRNA and protein expression of BAMBI. The results were represented as mean ± SD; *n* = 3; * *p* < 0.05; ** *p* < 0.01.

In order to investigate the relationship between BAMBI and Wnt/β-catenin signaling transcription activity in C2C12 cells, we measured *Axin2* transcription and the nuclear translocation of β-catenin, which are two indicators for Wnt/β-catenin activity. At 48 h post induction, *BAMBI* siRNA decreased these two indicators significantly (Figure 3a–c). Subsequently, we activated Wnt/β-catenin signaling using 10 mM LiCl, and found that LiCl restored the influence of BAMBI on Wnt/β-catenin signaling (Figure 3d–f) but did not alter the expression of *BAMBI* (Figure 3g,h).

### 2.4. LiCl Rescued the Influence of BAMBI Interference on C2C12 Differentiation

**Figure 4:**
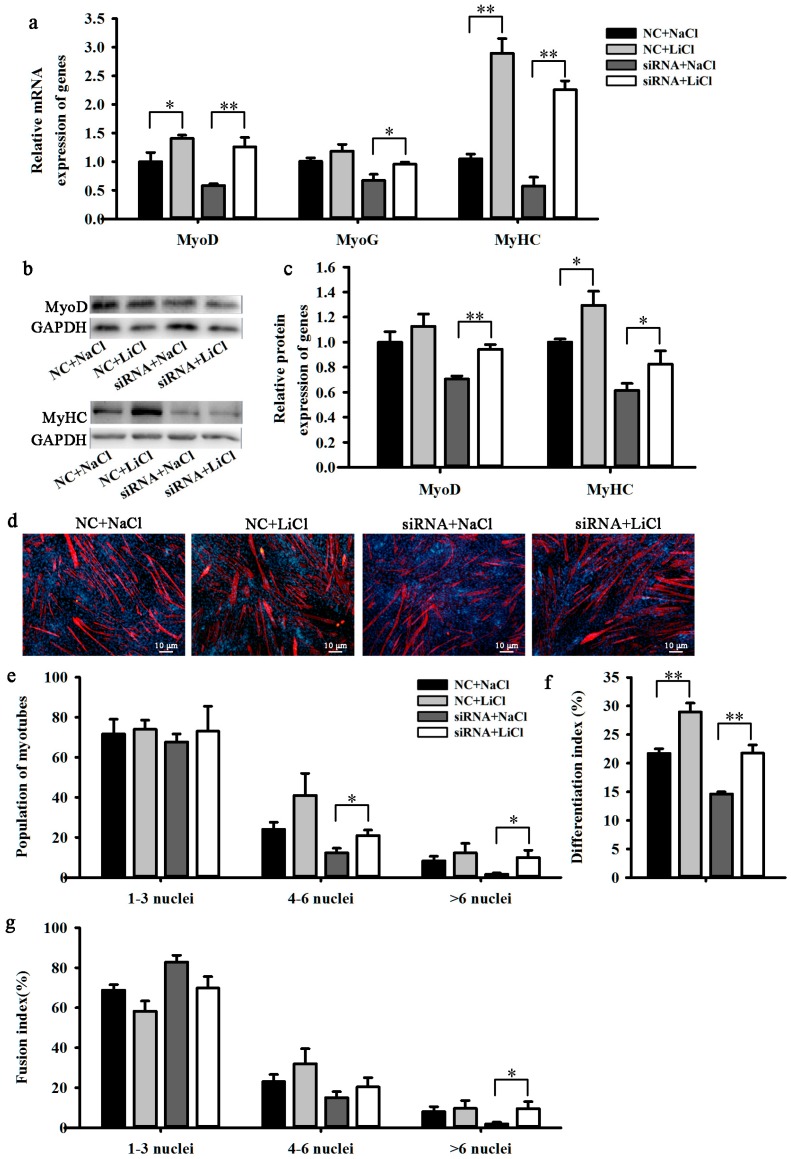
LiCl rescued the inhibitory effect of *BAMBI* siRNA on C2C12 myogenic differentiation. All the cell samples were harvested after transfection and myogenic induction for 48 and 96 h. (**a**) The mRNA expression of *MyoD* at 48 h and that of *MyoG* and *MyHC* at 96 h; (**b**) the western blot images of *MyoD*, *MyHC*, and *GAPDH*; (**c**) the protein expression of *MyoD* at 48 h and *MyHC* at 96 h; (**d**) immunofluorescence images of *MyHC* in C2C12 myotubes at 96 h post differentiation, images captured at 100× magnification; (**e**) the populations of myotubes; (**f**) the differentiation index and (**g**) the myotube fusion index. The results were represented as mean ± SD; *n* = 3; * *p* < 0.05; ** *p* < 0.01.

The expression of *BAMBI* was reduced significantly by treatment with siRNA in media containing 10 mM LiCl or NaCl (Figure 3g,h). In the presence of NC, LiCl promoted the expression of *MyoD*, *MyoG*, and *MyHC* compared with their expression in cells treated with NaCl (Figure 4a–c). The population of myotubes and the differentiation and fusion indices were increased significantly (Figure 4d–g). In the presence of siRNA, LiCl rescued the negative influence of the knockdown of BAMBI on the above indicators (Figure 4). These results suggest that BAMBI promotes C2C12 myogenic differentiation by enhancing Wnt/β-catenin signaling.

## 3. Discussion

Skeletal muscle development is a highly orchestrated biological process, involving myogenic progenitor cell maintenance, lineage specification, proliferation, and terminal differentiation. Wnt signaling participates in the regulation of embryonic muscle development and adult muscle regeneration [15,16]. During the onset of C2C12 myogenic differentiation, the expression of *BAMBI* was strongly induced prior to that of *MyoD*, and then it gradually declined. Knockdown of *BAMBI* using siRNA impaired the expression of *MyoD*, *MyoG*, and *MyHC* and the formation of myotubes, indicating that BAMBI plays an essential role during C2C12 myogenic differentiation.

Canonical Wnt proteins mainly promote myogenic lineage specification, mainly via inducing the activity of myogenic regulatory factors by way of the β-catenin/T-cell factors (TCFs) transcriptional complex [17]. Wnt3a contributes to the post-translational regulation and activation of *MyoD* and *MyoG* during myogenesis in P19 embryonal carcinoma stem cells [18]. *MyoD* is a direct target of Wnt/β-catenin in myogenesis [19]. Nuclear β-catenin interacts with *MyoD* and enhances its binding ability to the E-box element and its transcriptional activity [20].

It is well known that BAMBI exerts its functions through several signaling pathways, such as blocking TGF-β/Smad [7] and activating Wnt/β-catenin or extracellular signal-related kinase 1/2 signaling [5,21]. BAMBI facilitates the interaction between Frizzled5 and Dishevelled2, thus promoting canonical Wnt activity [8]. We also found that the experimental reduction of *BAMBI* expression significantly inhibited the nuclear translocation of β-catenin and the transcription of *Axin2*, two indicators of Wnt/β-catenin activity [22,23,24]. Similar results were observed in gastric cancer cells and preadipocytes [3,5,6]. The inhibitory effects of BAMBI knockdown on myogenic differentiation and Wnt/β-catenin signaling were reversed by supplementation with LiCl, an activator of Wnt/β-catenin activity [25,26]. These results show that Wnt/β-catenin signaling is a mediator of BAMBI’s role during C2C12 myogenic differentiation.

Notably, we did not observe any change in *BAMBI* expression following the treatment of C2C12 cells with LiCl, in accordance with previous results in Simpson–Golabi–Behmel syndrome preadipocytes [6], although *BAMBI* was previously identified as a downstream target of Wnt/β-catenin signaling in several other cell types [5,27]. These discrepancies may be due to differences among the cell models studied.

To further elucidate the mechanisms of BAMBI’s functions in regulating myogenic differentiation, direct knockdown of TCFs or specific inhibitors against the physical interaction between β-catenin and TCFs may be among the ideal methods to use in future research. Wnt signaling represents a gigantic regulatory network that consists of more than 40 components, and the relationship between myogenesis and Wnt signaling is far from being fully elucidated [15,16]. The ligands for Wnt signaling, such as Wnt3a, may act in a dose-dependent manner on C2C12 differentiation [24,28,29]. Nonetheless, the β-catenin/TCFs complex has unambiguous functions in C2C12 differentiation.

## 4. Experimental Section

### 4.1. Cell Culture

C2C12 cells [30] (ATCC, Manassas, VA, USA) were grown in DMEM (Invitrogen, Carlsbad, CA, USA) medium supplemented with 10% (*v*/*v*) fetal bovine serum (Hyclone, Logan, UT, USA), 2 mM L-glutamine, and 1% (*v*/*v*) penicillin/streptomycin solution at 37 °C and 5% CO_2_. When the cell density reached 90%, the growth medium was replaced with differentiation medium, which was DMEM medium containing 2% (*v*/*v*) horse serum (Invitrogen), 2 mM L-glutamine, and 1% (*v*/*v*) penicillin/streptomycin solution. All the used cells were at similar passages (P5–7). Media were changed every two days. LiCl (Solarbio, Beijing, China) was diluted to 1 M using ultrapure water.

### 4.2. Transfection of BAMBI siRNA and the siRNA Negative Control

*BAMBI* siRNA and a scrambled negative control (NC) were designed and purchased from Invitrogen (Table 1). When the cell density reached 70%, the C2C12 myoblasts were subjected to starvation in Opti-MEM^®^ I reduced serum medium (Invitrogen) for 4 h prior to transfection. Lipofectamine^®^ 2000 transfection reagent (Invitrogen) and siRNA or NC were gently mixed according to the manufacturer’s protocol, and then added to the culture supernatant. After 6 h of incubation, Opti-MEM I medium was replaced with fresh DM.

**Table 1:** siRNA targeting the coding region of mouse *BAMBI*.

siRNA	Sequences (5′–3′)	
*BAMBI* siRNA	Sense	GCAAGCAGAGCUCAGUAAUTT
	Antisense	AUUACUGAGCUCUGCUUGCTT
Negative control	Sense	GCAAGCAGAGCUCAGUAAUTT
	Antisense	ACGUGACACGUUCGGAGAATT

### 4.3. Real-Time Quantitative PCR

Trizol^®^ reagent (TaKaRa Bio, Otsu Japan) was applied to extract cellular total RNA. The RNA quality and concentration were estimated using agarose gel electrophoresis and spectrophotometry (Nanodrop, Wilmington, DE, USA), respectively. The total RNA was processed into single stranded cDNA using a reverse transcription kit (TaKaRa Bio). Real-time quantitative PCR was performed using a SYBR^®^ green kit (TaKaRa Bio) on a Bio-Rad iQTM5 system (Bio-Rad, Hercules, CA, USA). *GAPDH* was used as a housekeeping gene for normalizing the expression of other genes. The 2^−∆∆*C*t^ algorithm was employed to estimate the relative expression level of each gene. The sequences of primers can be found in Table 2.

### 4.4. Western Blot Detection

Total and nuclear proteins of C2C12 cells were extracted using kits from Vazyme, Nanjing, China. The protein concentration was determined using the bicinchoninic acid assay kit (Vazyme), and then 5× protein loading buffer was added to the lysates prior to full denaturation in boiled water for 10 min. A total of 20 µg of protein was electrophoresed on a 12% SDS-polyacrylamide gel and transferred to a polyvinylidene difluoride membrane (Cell Signaling Technology, Danvers, MA, USA). The membrane was blocked in 5% (*w*/*v*) defatted milk at room temperature for 2 h, and then incubated at 4 °C overnight with primary antibodies, including MyoD (1:300 in Tris-buffered-saline containing Tween^®^ 20 (TBST), sc-760, Santa Cruz Biotechnology, Dallas, TX, USA), BAMBI, MyHC (AF2387, MAB4470, 1:400 in TBST, R&D Systems, Minneapolis, MN, USA), β-catenin (1:400 in TBST, bs-1165R, Bioss, Beijing, China), laminB1 (ab16048, 1:1000 in TBST, Abcam, Cambridge, UK), and GAPDH (1:400 in TBST, BA2913, Boster, Wuhan, China). The next day, the membranes were washed with TBST and incubated with goat anti-mouse or anti-rabbit secondary antibodys (BA1050, BA1054, 1:1000 in TBST, Boster, Wuhan, China) at room temperature for 2 h. The membranes were washed three times with TBST and then exposed using a ChemiDoc XRS imaging system (Bio-Rad). The captured images were analyzed by Quantity One 4.6.3 software (Bio-Rad). The protein level of whole cell lysates was normalized against the expression of GAPDH, whereas the protein level of nuclear lysates was normalized against the expression of laminB1.

**Table 2:** Specific primers for real-time PCR.

Gene	Sequences (5′–3′)	Accession No.
*MyoD*	F: CATTCCAACCCACAGAACCT	NM_010866.1
	R: CAAGCCCTGAGAGTCGTCTT	
*MyoG*	F: CAATGCACTGGAGTTCGGT	NM_031189.2
	R: CTGGGAAGGCAACAGACAT	
*MyHC*	F: CGCAAGAATGTTCTCAGGCT	NM_030679.1
	R: GCCAGGTTGACATTGGATTG	
*Axin2*	F: CGCTCGGGTTTGTGTTAAGT	NM_015732.4
	R: GTCAACGCTCTGCCCTACAC	
*BAMBI*	F: AAGCCTCAGGACAAGGAAA	NM_026505.2
	R: CAATGGGAACCGCTATCA	
*GAPDH*	F: AACTTTGGCATTGTGGAAGG	NM_008084.3
	R: ACACATTGGGGGTAGGAACA	

### 4.5. Immunocytochemical Analysis

Four days post myogenic differentiation, C2C12 cells were washed with cold PBS and fixed with 4% (*w*/*v*) paraformaldehyde for 15 min. 0.5% (*v*/*v*) Triton™ X-100 was used for permeabilization. The cells were then blocked in 0.5% (*w*/*v*) bovine serum albumin diluted in PBS. After blocking, the cells were incubated first with anti-MyHC antibody (1:250 in TBST, MAB4470, R&D Systems) at 37 °C for 2 h, and then with red fluorescence-labeled secondary antibody (a-11079, Invitrogen) at 37 °C for 1 h. The nuclei were stained with DAPI (1:1000 in PBS, 10236276001, Roche, Basel, Switzerland) for 10 min. Images were captured using a Nikon TE2000 microscope at 100× magnification (Nikon, Tokyo, Japan). The numbers of myotubes with 1–3, 4–6, and >6 nuclei were counted and averaged among three images per treatment at 100× magnification. The differentiation index was determined as the percentage of MyHC-positive nuclei among total nuclei, and the myotube fusion index was determined as the distribution of the nucleus number in total myotubes [12].

### 4.6. Statistical Analysis

Statistical analyses were performed in SPSS 19.0 statistical software (IBM, Armonk, NY, USA) using the Student *t*-test. Data are expressed as the mean ± SD. Statistical significance was indicated as follows: * *p* < 0.05; ** *p* < 0.01.

## 5. Conclusions

Our studies identify that the BAMBI is an essential and positive regulator of C2C12 differentiation, and Wnt/β-catenin signaling mediates its role during myogenic differentiation.
